# The effect of acupuncture at the Yuji point on resting-state brain function in anxiety

**DOI:** 10.1097/MD.0000000000033094

**Published:** 2023-02-22

**Authors:** Yuangeng Wang, Chunlin Li, Xianghua Qi

**Affiliations:** a First College of Clinical Medicine, Shandong University of Traditional Chinese Medicine, Jinan, China; b Department of Encephalopathy, Affiliated Hospital of Shandong University of Traditional Chinese Medicine, Jinan, China.

**Keywords:** anxiety, fMRI, functional connectivity, Yuji point

## Abstract

**Background:**

The COVID-19 epidemic has placed a lot of mental burdens on school students, causing anxiety. Clinically, it has been found that the Yuji point (LU10) can relieve anxiety by regulating Qi.

**Methods:**

Thirty-six volunteers with anxiety disorders were divided into 3 groups, all of whom underwent 2 MRI examinations. The Yuji and nonacupoint groups received acupuncture between functional magnetic resonance imagings. We used the amplitude of low-frequency fluctuation to analyze regional brain activity, and seed-based functional connectivity (FC) to analyze changes in brain networks.

**Results:**

After acupuncture, the LU10 was able to activate the frontal lobe, medial frontal gyrus, anterior cingulate gyrus, temporal lobe, hippocampus, etc in the left brain compared to the control group. The frontal lobe, medial frontal gyrus, cingulate gyrus, and anterior cingulate gyrus in the left brain were activated compared to those in the nonacupoint group. Compared with the control group, LU10 showed increased FC in the right parietal lobe, right precuneus, left temporal lobe, left superior temporal gyrus, and with cingulate gyrus. FC was enhanced among the hippocampus with the left temporal lobe and the superior temporal gyrus and reduced in the right lingual gyrus and right occipital lobe.

**Conclusion:**

Acupuncture at LU10s can regulate anxiety by upregulating or downregulating the relevant brain regions and networks. LU10s can be used to treat not only lung disorders but also related mental disorders.

## 1. Introduction

Anxiety is defined as “worried anticipation of future danger or misfortune, accompanied by somatic symptoms of anxiety or stress.“ The focus of the expected hazard may be internal or external.^[1]^ Anxiety can be expressed in several ways.^[1]^ Subjective feelings, such as uneasiness and nervousness, are often accompanied by somatic symptoms such as dizziness, sweating, trembling, and panic. Occasional and transient anxiety are appropriate responses to the external environment and can help people avoid danger and adapt to change. Frequent or chronic anxiety can lead to pathological changes.^[2]^

The prevalence of anxiety is influenced by cultural contexts and background environments. Medical students spend long periods in school and are under academic stress. In a systematic review including 21 surveys, 35160 Chinese medical students, the mean prevalence of anxiety disorders was 27.22%.^[3]^ In addition to the influence of the family environment, poor interpersonal relationships, academic stress, low interest and satisfaction with their current profession, poor self-evaluation, poor expectations of a future career, and lack of sleep increased the odds of anxiety.^[3]^ Since the outbreak of COVID-19, the continued spread of the epidemic and strict closure policies have impacted the psychological well-being of college students. Cao et al^[4]^ investigated the anxiety of 7143 Chinese medical students and found that 24.9% of college students suffered from anxiety disorders, of which 0.9% had severe anxiety and 21.3% had mild anxiety.

The Yuji point (LU10) belongs to the Taiyin lung meridian. It was located on the lateral side of the hand, at the midpoint of the first metacarpal radius, and at the junction of red and white skin. In the theory of “5 acupoints,” it belongs to the Xing point of the Lung meridian and has the function of “unblocking the Qi of the Lung meridian and expelling the actual evil in the lung.“ Therefore, it is often used to treat coughs, asthma, and other lung diseases. In Chinese medicine, when a person encounters a bad situation, stress, or persistent bad mood, it can lead to stagnation of Qi, resulting in a series of pathological changes and psychological problems, such as anxiety and depression. The liver is the master of emotions and governing conveyance and dispersion, the lung is the master of Qi, with the role of propagation and purification of Qi. All Qi in the body depends on the lung, and liver Qi is also dependent on the lung for its promotion and regulation. Li believes that Qi depression or lack of lung Qi can easily lead to depression due to lack of lung Qi, chest tightness, shortness of breath, palpitations, and other manifestations.^[5]^ The Yuji point (LU10) is the most prominent among the acupoints of the Hand Taiyin Lung meridian for regulating Qi in the chest and relieving depression. Sun applied the LU10 to treat insomnia of the liver-depression-Qi stagnation type and obtained good results.^[6]^

Functional magnetic resonance imaging (fMRI) can reflect temporal changes in metabolic regions of the brain. These changes may be due to task-induced changes in the cognitive state or unmodulated processes in the resting brain.^[7]^ The principle is to observe changes in the ratio of oxygenated to deoxygenated hemoglobin based on blood oxygen level dependence.^[7]^ Reduced functional brain connectivity between the amygdala and certain cortical regions has been observed in patients with bipolar affective disorder undergoing resting-state fMRI.^[8]^ The application of fMRI can reflect the immediate and sustained effects of acupuncture. In a systematic evaluation of the effects of acupuncture on functional brain networks, acupuncture points increased the connectivity of the default mode network and sensorimotor network with pain, emotion, and memory-related brain areas. Acupuncture increased connectivity between the periaqueductal gray matter, anterior cingulate cortex, left posterior cingulate cortex, right anterior insula, limbic/paracentral, and precuneus lobes. Reduced connectivity is mainly associated with the sensory cortex and subcortical areas. Acupuncture is used to treat Alzheimer disease and hypocarbia by modulating the connectivity of the limbic/parameningeal neocortical network, brainstem, cerebellum, subcortical, and hippocampal brain regions.^[9]^

In previous research work, there has been less clinical research on the Yuji point, which is a common point in our treatment of anxiety and depression. We hypothesized that the use of resting MRI studies would reflect the effects of acupuncture of the LU10 on anxious patients through alterations in the function and connectivity of brain areas. Consequently, to investigate the neural mechanisms by which acupuncture LU10 ameliorates anxiety symptoms, we used fMRI. Relevant brain regions were analyzed using the amplitude of low-frequency fluctuation (ALFF) data. In addition, changes in the functional connectivity of the brain were studied to explore the effects of acupuncture at LU10s on brain networks in anxious subjects.

## 2. Materials and Methods

### 2.1. Subjects

Thirty-six right-handed postgraduate students aged 20 to 35 years from Shandong University of Traditional Chinese Medicine. All the subjects with anxiety disorders signed an informed consent form. In compliance with the Declaration of Helsinki, this study was approved by the Ethics Review Committee of the Affiliated Hospital of Shandong University of Traditional Chinese Medicine ([2021], Ethics Approval No. [014]-KY). The registration number is ChiCTR2200061607. Using the computer random number table method, 36 subjects were randomly divided into 3 groups with 12 subjects in each group. The control group did not receive acupuncture treatment, and the Yuji group received acupuncture treatment at the Yuji point. The needles in the nonacupoint group were nonmeridians and nonacupuncture points. The blinding method was used only for the subjects, blinding was unmasked after the start of needling.

### 2.2. Diagnostic criteria

The anxiety status of all volunteers was scored by trained professionals using the Self-Assessment Scale for Anxiety. Anxiety is defined as when the Self-Assessment Scale for Anxiety score is > 50, with 50 to 59 being mild anxiety, 60 to 69 being moderate anxiety and 69 or more being severe anxiety. A psychiatrist made the diagnosis of an anxiety disorder ([ICD-10/F41]). The specific diagnostic criteria were based on the anxiety diagnostic criteria of the Chinese Classification and Diagnostic Criteria of Mental Disorders (Third Edition) ([CCMD-3]) published in 2001. The inclusion and exclusion criteria for the study participants are shown in Table 1.

**Table 1:** Inclusion and exclusion criteria.

Inclusion criteria:
(1) 20 to 35 yr old
(2) right-handed
(3) anxiety disorders
(4) SAS score>50
(5) Sign the informed consent form
Exclusion criteria:
(1) with major physical diseases
(2) contraindications for fMRI detection
(3) with an acupuncture experience in the past w
(4) Used medication in the last 2 w

fMRI = functional magnetic resonance imaging, SAS = Self-Assessment Scale for Anxiety.

### 2.3. Intervention procedure

**Figure 1.**
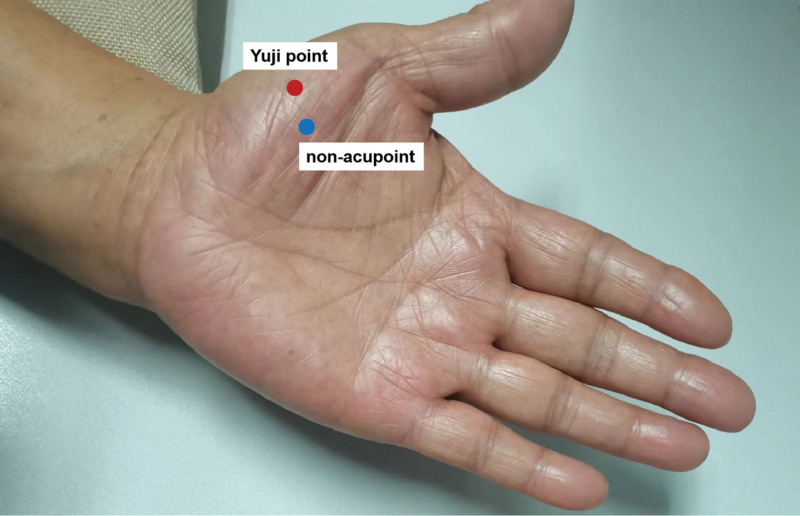
Diagram of acupoints.

**Figure 2.**
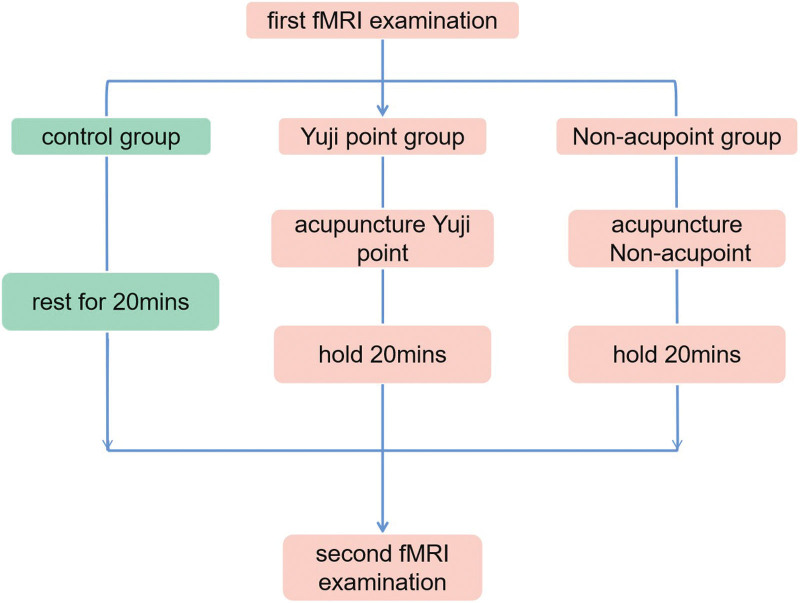
Research process.

All 3 groups of volunteers underwent twice fMRI examinations. The Yuji group was an acupunctured LU10 (Fig. 1) and held it for 20 minutes after De Qi, and the nonacupoint group was an acupunctured nonacupoint (Fig. 1) and held it for 20 minutes. The control group was allowed to rest for 20 minutes. fMRI was performed again in all 3 groups after the completion of acupuncture. This process is illustrated in Figure 2.

Prior to the start of the MRI examination, 36 subjects received instructions related to the examination to eliminate fear and reduce interference with the experiment. The contents included that the functional MRI examination was performed in a relatively closed environment with a lack of air circulation and various noises, which might cause discomfort to the subjects, and that the subjects should be prepared in advance. We provided the subjects with a comfortable ambient temperature and noise-resistant earplugs (3M, USA) to reduce noise interference. Additionally, a head coil with foam was used to immobilize the heads of the subjects to reduce head movement. During functional MRI, the subjects were asked to remain still and avoid emotional activity. Each fMRI examination took approximately 20 minutes, and the subjects withdrew from the experiment when they felt unable to tolerate fMRI or when acupuncture had adverse reactions such as dizziness.

### 2.4. Acupuncture manipulation points

We chose acupuncture needles measuring 40 mm × 0.40 mm (Wujiang City Yunlong Medical Equipment Co.). Acupuncture was manipulated by a professional acupuncturist with more than 10 years of practical experience in acupuncture. All acupoints were selected from the left hand. The LU10 was identified according to an internationally standardized protocol. The nonacupoint was located 1 cm from the medial side of LU10. Routine alcohol disinfection was followed by needling up to a depth of 1 cm. Manipulation was performed to stimulate De Qi, with a twisting amplitude usually around 180° to 360°. The elevation and insertion amplitude were 3 to 5 mm, and the frequency was 100 to 150 times/minute. De Qi is a state in which needling takes effect, caused by the induction of meridian Qi at the site of needling. The specific manifestations are: after needling the acupuncture point, following manipulation or longer needle retention, the patient experiences soreness, numbness, swelling, heaviness, and other sensations; the acupuncturist feels the needle sink and tighten. The presence or absence of Qi is closely related to the effectiveness of the needling. The sensations of the De Qi state include pain, numbness, swelling, and tenderness, subjects will be asked if they feel any of the above to determine if they are in the De Qi state. The needles were maintained for 20 minutes after De Qi. The acupuncture was performed in a separate room. The needles were checked and inspected after completion to prevent the patient from bringing them into the MRI room.

### 2.5. fMRI examinations

All fMRI examinations were performed at the Affiliated Hospital of the Shandong University of TCM. The fMRI scanner was a 3.0 T whole-body Achibia MRI scanner with an 8-channel RF coil. fMRI images were obtained using gradient echo-planar imaging (GRE-EPI; TR/TE/FA, 3000 mm/35 mm/90°; FOV, 230 mm × 230 mm; slice thickness/gap/gap 5.0 mm/0 mm; matrix, 128 × 128; interlayer scan).

### 2.6. Data preprocessing and calculating

We used a configurable pipeline for analysis of connectomes for data processing and the NeuroScholar cloud platform (http://www.humanbrain.cn, Beijing Intelligent Brain Cloud, Inc.) for acceleration and simplification, which is a python-based pipeline tool involving AFNI,^[10]^ ANTs,^[11]^ FSL,^[12]^ and custom Python code to process the data, and is available on https://fcp-indi.github.com. We performed structural processing of the brain images, including position calibration, skull stripping, tissue segmentation, and the Montreal Neurological Institute 152 stereotactic space (1 mm isotropic) for spatial normalization. In functional preprocessing, the first 10 time points were removed, slice time correction, image position correction; motion correction to obtain motion parameters; bandpass time filtering from (0.01 Hz–0.08 Hz; 11) z-score followed by smoothing (FWHM = 6.0 mm); cranial separation and tissue segmentation, and registration of functional images to anatomical space; motion artifacts were removed using ICA-AROMA and partial component regression.^[13]^ Quality control should exclude subjects who: contain motion artifacts or excessive noise; and have an organic brain disease and structural asymmetry. Nuisance signal regression was applied, including mean values of white matter and cerebrospinal fluid signals, 24 motion parameters, and linear trends in the time series, and no subjects were excluded due to noncompliant image quality.

## 3. Analysis

Measurements between groups were compared using the Q test and are described as mean ± standard deviation (±s). The count data were compared using the χ2 test. p is a 2-sided test with no significant differences at *P* > .05. Data were analyzed using SPSS 21.0.

The analysis was performed using the statistical tools in DPABI,^[14]^ ANOVA to generate ALFF,^[15]^ and comparing the 3 groups. Scheffe post hoc analysis was also conducted. Gaussian Random Field (GRF) correction with voxel-wise *P* < .001 and cluster-wise *P* < .05 was used for multiple comparison correction. Regions showing significant results in the ALFF were defined as the ROIs. Thus, seed-based whole-brain functional connectivity analysis (SCA) was performed using these ROIs. functional connectivity (FC) maps from SCA were forwarded for a 2-sample *t* test between groups.

## 4. Results

### 4.1. Subjects baseline data

Subjects baseline information is shown in Table 2.

**Table 2:** Subjects baseline information.

	Sex (Male/Female)	Average age	Average SAS score
Control group	3/9	26.25	64.67
Yuji group	1/11	25.17	66.4
Nonacupoint group	3/9	24.42	57.7

SAS = Self-Assessment Scale for Anxiety.

### 4.2. ALFF results

**Figure 3.**
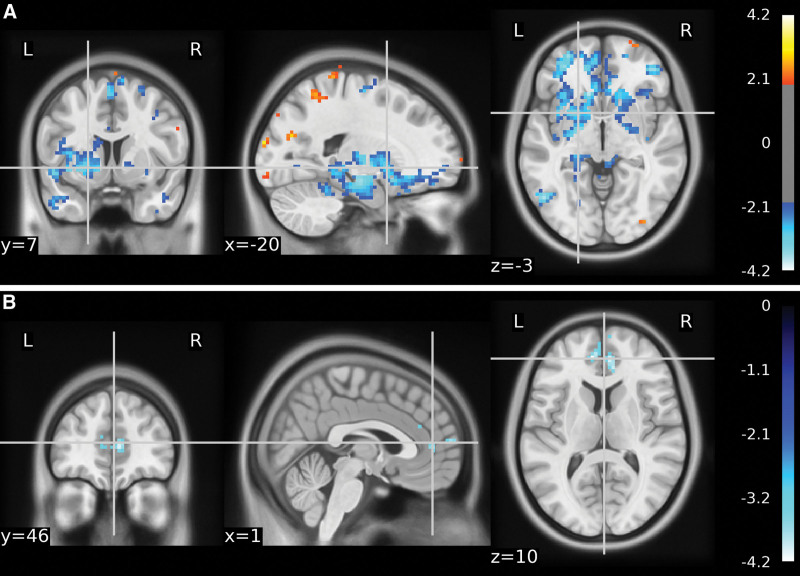
The ALFF values of the control group were analyzed in comparison with the Yuji group to detect significant changes in brain areas. *P* < .001. brain areas with decreased ALFF values are in blue color. (A) Deactivation of brain regions in the control group compared with the Yuji group. Left frontal lobe/left internal frontal gyrus/left ACC.(B) Deactivated brain regions in the control group compared with the Yuji group. Left temporal lobe/left inferior gyrus/hippocampus. ACC = anterior cingulate gyrus, ALFF = amplitude of low-frequency fluctuation.

Data from all 36 participants were included in the analysis. In the initial fMRI results, no significant differences were found between the 3 groups. The total functional volume in the control group compared to the Yuji group was 189. Compared to the control group, the functional MRI results of the subjects in the Yuji group showed positive activation in the left frontal lobe, left medial frontal gyrus, left anterior cingulate gyrus, left temporal lobe, left subgyral, and left hippocampus (Fig. 3A and B). Table 3 shows the significant differences in the peak voxels within the different clusters. In contrast, there were no differences in the brain areas between the control and nonacupoint groups.

**Table 3:** Differential brain regions in the Control group versus the Yuji group.

Brain regions		Voxels	Peak intensity (*T* value)	MNI coordinates		
				*X*	*Y*	*Z*
Deactivated regions	Left frontal lobe left medial frontal gyrus left anterior cingulate gyrus	112	−4.21742	−9	48	9
	Left temporal lobe left subgyral hippocampus	77	−4.22298	−24	−18	−12

MNI = Montreal Neurological Institute.

**Figure 4.**
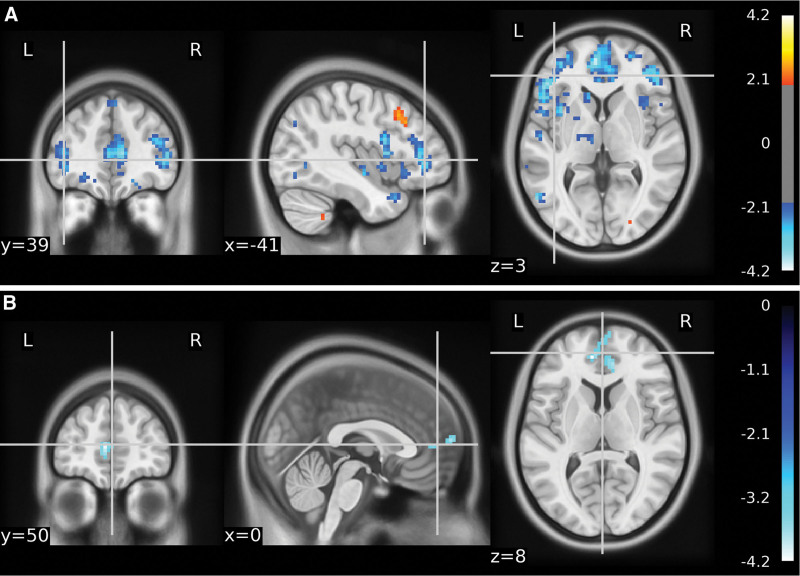
The ALFF values of the nonacupoint group were analyzed in comparison with the Yuji group to detect significant changes in brain areas. *P* < .001. Blue color represents brain areas with decreased ALFF values. (A) & (B) Brain regions deactivated in the nonacupoint group versus the Yuji group. Left frontal lobe/left medial/frontal gyrus/left glial brain/left ACC. ACC = anterior cingulate gyrus, ALFF = amplitude of low-frequency fluctuation.

The total functional volume in the nonacupoint group compared to the Yuji group was 92. Activation of the left frontal lobe, left medial frontal gyrus, left cingulate, and left anterior cingulate gyrus (ACC) was found in the yuji group compared to the nonacupoint group (Fig. 4A and B, Table 4).

**Table 4:** Differential brain regions in nonacupoint group versus Yuji group.

Brain regions		Voxels	Peak intensity (*T* value)	MNI coordinates		
				X	Y	Z
Deactivated regions	Left frontal lobe left medial frontal gyrus left cingulum left anterior cingulate gyrus	92	−4.20822	−9	48	9

MNI = Montreal Neurological Institute.

### 4.3. FC analysis results

**Figure 5.**
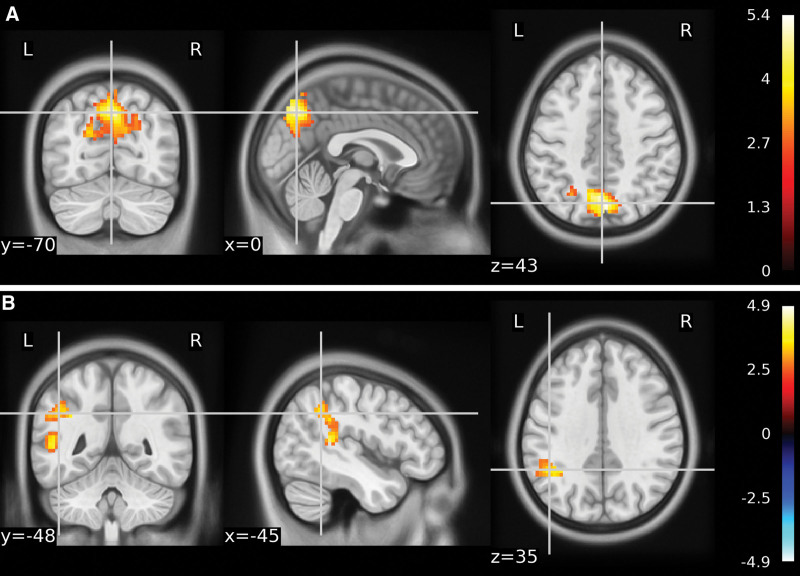
Functional connectivity in the Yuji group versus the control group. *P* < .01. Red indicates an increase in regional FC and blue indicates a decrease. (A) Cingulate gyrus shows increased FC with the right parietal, right precuneus, left temporal lobe, and left superior temporal gyrus. (B) Hippocampus shows increased FC to the left temporal lobe and superior temporal gyrus and decreased FC to the right lingual gyrus and right occipital lobe. FC = functional connectivity.

**Figure 6.**
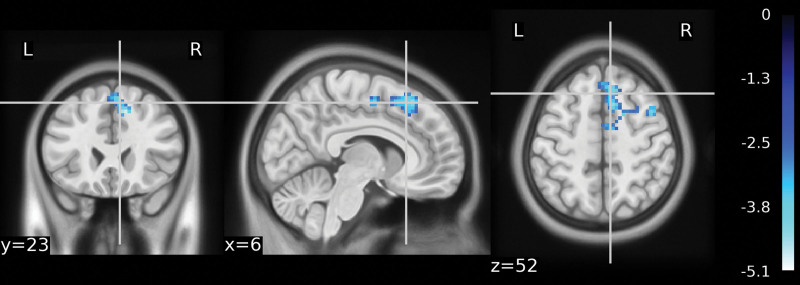
Functional connectivity in the nonacupoint group compared to the Yuji group. The FC between the medial frontal gyrus and the right frontal lobe was reduced in the nonacupoint group versus the Yuji group. FC = functional connectivity.

To analyze the functional connectivity of positive and negative activated regions with other brain regions between different groups, regions showing significant results in the ALFF were defined as ROIs. Therefore, SCA was performed using these ROIs. A sample t-test was used for group comparisons of FC. The cingulate gyrus showed increased FC in the right parietal, right precuneus, left temporal lobe, and left superior temporal gyrus in the Yuji group compared with the control group. The hippocampus showed increased FC in the left temporal lobe and superior temporal gyrus, while decreased FC in the right lingual gyrus and right occipital lobe (Fig. 5A and B, Table 5). Compared to the nonacupoint group, we observed an increase in FC of the right frontal lobe and the medial frontal gyrus to the cingulum in the Yuji group (Fig. 6, Table 6).

**Table 5:** Functional connectivity (FC) analysis based on seed points of differential brain regions between the Control group and the Yuji group.

Seeds	Decreased FC regions	Increased FC regions	Voxels	Peak intensity (*T* value)	MNI coordinates		
					X	Y	Z
Cingulum		Right parietal lobe right precuneus	652	5.37146	3	−72	45
Hippocampus		Left temporal lobe left superior temporal gyrus	226	4.15123	−45	42	12
	Right occipital lobe right lingual gyrus		130	−4.91544	6	84	−6

FC = functional connectivity, MNI = Montreal Neurological Institute.

**Table 6:** Functional connectivity (FC) analysis based on seed points of differential brain regions between the Yuji group and nonacupoint group.

Seeds	Decreased FC regions	Increased FC regions	Voxels	Peak intensity (*T* value)	MNI coordinates		
					X	Y	Z
Cingulum	Right frontal lobe medial frontal gyrus	/	288	−5.07059	12	27	45

FC = functional connectivity, MNI = Montreal Neurological Institute.

## 5. Discussion

In our study, we used fMRI analysis to investigate the mechanism of action of acupuncture at Yuji in alleviating anxiety. The ALFF results showed positive activation in the left frontal lobe, left medial frontal gyrus, left anterior cingulate gyrus, left temporal lobe, left subgyral, and hippocampus when acupuncture was performed in LU10 compared to the control group. The prefrontal and cingulate cortex, amygdala, striatum, hippocampus, and thalamus regions have been shown to be associated with anxiety and depression.^[16]^ In our previous study, acupuncture at the Neiguan point in patients with anxiety disorders was able to positively activate the left parahippocampal gyrus, cingulate gyrus, and right superior temporal gyrus, and negatively activate the right middle frontal gyrus, right precuneus, and cuneus regions.^[17]^

The frontal lobe is the higher center that controls human behavior, thoughts, and emotions. The ACC and the medial prefrontal cortex (mPFC) comprise the medial wall of the frontal lobe. The cingulate gyrus is the brain gyrus located between the cingulate sulcus and corpus callosum sulcus on the medial side of the cerebral hemisphere and belongs to the cortical part of the limbic system. ACC plays an important role in emotional responses and sensory perception.^[18]^ Etkin et al^[19]^ suggested that the dorsal caudal region of the ACC/mPFC is associated with the assessment and expression of negative emotions, whereas the ventral portion of the ACC/mPFC modulates limbic regions involved in the generation of emotional responses. In anxious patients, the activity of the ACC is altered, and its severity is positively correlated with the right parahippocampal region and the left anterior cingulate region.^[20]^ In animal models of chronic pain and anxiety, inhibition of ACC synaptic plasticity has been shown to produce analgesic and anxiolytic effects.^[21,22]^ In chronically inflamed rats, pain can lead to anxiety-like behavior. Electroacupuncture suppresses pain and anxiety-like behaviors in rats by enhancing the expression of the NPS/NPSR system in the ACC.^[23]^ Zhao et al reported electroacupuncture stimulation in patients with irritable bowel syndrome and anxiety and observed a decrease in ALFF values in the ACC and PFC.^[24]^

The temporal lobe is critical for cognitive processes, including emotion, memory, and language processing, with the anterior part of the temporal lobe being the mental cortex, which is closely related to emotion. The hippocampus, located between the cerebral thalamus and medial temporal lobe, is part of the limbic system and is primarily responsible for functions such as storage, conversion, and orientation of short-term memory. The ventral hippocampus may play a preferential role in brain processes associated with anxiety-related behaviors.^[25]^ Defects in hippocampal neurogenesis have been suggested to lead to affective and anxiety disorders,^[26]^ and this has been confirmed in rodents.^[27]^ In response to alginate (KA)-induced seizures and neurodegeneration of hippocampal CA3 pyramidal cells in mice, acupuncture inhibits hippocampal cell death.^[28]^

No differential brain regions were found between the nonacupoint and the control groups. Compared with the nonacupoint group, the Yuji group showed an elevated ALFF in the left frontal lobe, left internal frontal gyrus, left cingulate, and left ACC. Therefore, we suggest that the improvement in anxiety symptoms by acupuncture in Yuji may be related to activation in the left internal frontal gyrus, left anterior cingulate gyrus, and left hippocampus.

In addition, a functional analysis of the changes in the associated brain networks induced by acupuncture was performed. The results showed increased FC of the cingulate gyrus with the right parietal, right precuneus, left temporal, and left superior temporal gyri in Yuji compared with the control. The hippocampus showed elevated functional connectivity with the left temporal lobe and superior temporal gyrus, while decreased connectivity with the right lingual gyrus and right occipital lobe. The default mode network (DMN) includes the posterior cingulate cortex, precuneus, medial prefrontal cortex, inferior parietal lobule, hippocampus, and bilateral temporal cortex.^[29]^ In patients with anxiety disorders, the DMN is regulated by both 5-Hydroxytryptamine and noradrenergic modulation; when the activity of both is reduced, the connectivity of the DMN decreases.^[29]^ North off argued that resting-state functional connectivity studies suggest that the DMN is abnormal in all anxiety disorders.^[30]^ In our study, acupuncture at the LU10 improved DMN connectivity and alleviated anxiety symptoms. Patients with bipolar disorder show reduced gray matter concentrations in the parietal-occipital-cerebellar network, with lower concentrations associated with higher self-reported impulsivity.^[31]^ Couvy-Duchesne et al^[32]^ found a reduction in gray matter surface area in the partial right lingual, sinohypoglossal, and parietal gyri in patients with anxiety depression. Thus, there is a link between the lingual gyrus and occipital lobe and psychological problems such as anxiety. In our study, we found that acupuncture of the LU10 decreased the connectivity of the hippocampus with the occipital lobe and lingual gyrus which is subject to further explanation.

We observed increased FC between the cingulate gyrus and the right frontal and intraparietal gyrus in the Yuji group versus the no-acupoint group. The cingulate gyrus and the right frontal and medial frontal gyrus belong to the executive control network, which is closely related to emotion regulation. Brain regions such as the ACC, amygdala, and medial prefrontal cortex have a reduced capacity for emotion regulation in patients with anxiety disorders, implying an important role of the prefrontal-limbic network.^[33]^ Compared with acupuncture at nonacupoints, acupuncture at Yuji seems to affect patients’ anxiety state by modulating the prefrontal-limbic network system.

## 6. Limitations

This study is about the immediately effect of acupuncture on changes in the functioning of brain regions in anxious participants. Patients with higher levels of anxiety require a longer intervention time, so a subsequent study design in this area is needed.

## 7. Conclusion

In conclusion, acupuncture at the LU10s altered the activity of several brain regions associated with the regulation of anxiety. This provides a basis for studying the brain mechanisms involved in the treatment of anxiety by acupuncture at the Yuji point, and provides support for traditional Chinese medicine to improve anxiety states by regulating Qi.

## Author contributions

**Conceptualization:** Yuangeng Wang.

**Funding acquisition:** Chunlin Li.

**Investigation:** Yuangeng Wang, Chunlin Li.

**Methodology:** Yuangeng Wang.

**Resources:** Chunlin Li.

**Supervision:** Xianghua Qi.

**Writing – original draft:** Yuangeng Wang.

**Writing – review & editing:** Xianghua Qi.
